# Estimation of the Expected Change in Domestic Human Salmonella Cases in Sweden in 2010, Given a Hypothetical Relaxation of the Current Salmonella Control Programme

**DOI:** 10.1371/journal.pone.0089833

**Published:** 2014-03-03

**Authors:** Helene Wahlström, Susanna Sternberg Lewerin, Kristian Sundström, Sofie Ivarsson

**Affiliations:** 1 Zoonosis Center, National Veterinary Institute, Uppsala, Sweden; 2 Department of Biomedical Sciences and Veterinary Public Health, Swedish University of Agricultural Sciences, Uppsala, Sweden; 3 Institute for Food and Agricultural Economics, Lund University, Lund, Sweden; 4 Department of Preparedness, Swedish Institute for Communicable Disease Control, Solna, Sweden; The Australian National University, Australia

## Abstract

The Swedish salmonella control programme has been very successful in reducing the number of salmonella infections in both humans and animals. However, the costs for the control have increased and it has thus been questioned if the control measures could be relaxed and, if so, what effect this would have on human and animal health. The aim of the present study is to evaluate the expected effects on human health of a relaxation of the Swedish control *i.e.* a substitution of the present programme with a programme similar to the ones present in Denmark or the Netherlands. Data from the year 2010 was used to illustrate this. It was assumed that the domestic exposure to salmonella would then become the same in Sweden as it was in Denmark or the Netherlands in that year. As official statistics on the number of reported salmonella cases are not comparable across European countries, data from five different sources were used to try to obtain comparable estimates of the domestic salmonella exposure in the three countries. The study shows that the number of reported domestic human salmonella cases in Sweden in 2010 would increase by approximately 900 to 2 400 cases in the Danish scenarios and 6 400 to 8 400 in the Dutch scenarios. Although uncertainty exists, it was concluded that the number of reported domestic salmonella cases would increase substantially in Sweden in case of a relaxation of the current control programme.

## Introduction

A severe outbreak of *S*. Typhimurium in Sweden (SE) in 1953 that involved more than 9 000 people prompted the need for a control programme for salmonella. Since then, the strategy for control has been to prevent salmonella in any part of the production chain. The current Swedish control programme covers the entire food chain from feed to food [1]. Any finding of salmonella in animals, animal products or feed is notifiable according to the Swedish law on zoonoses (Zoonoslagen, SFS 2006:1039), and measures to eliminate/eradicate salmonella are taken at any positive finding. Restrictions are put on infected holdings until they can be declared free from salmonella. The SE salmonella control programme has been very successful in reducing the number of salmonella infections in both animals and humans. The prevalence among SE food producing animals has remained below 0.5% for decades [2]. In humans, salmonellosis is notifiable according to the Communicable Disease Act (Smittskyddslagen, SFS 2004:168). About 600–800 domestic human cases of salmonellosis are reported every year, corresponding to an incidence of 6–8 cases/100 000 inhabitants [3]. The domestic cases constitute only about 20% of all reported cases. Except for Finland and Norway, this low proportion of domestic cases is unique from an international perspective [1].

An evaluation of the control programme was performed in 1993 where it was concluded that the control was cost effective [4]. However, since then the costs have increased and it has been questioned if the control measures could be relaxed and, if so, what effect this would have on human and animal health. The aim of the present paper was to evaluate what effect a relaxation of the SE programme would have on the number of domestic human cases of salmonella. As the current situation is considered dependent on the comprehensive national programme, it is not possible to evaluate the effect of changes of parts of the present programme. Instead, a scenario approach was used, where the expected effects of a hypothetical change from the current SE control programme to two other existing programmes were evaluated. The results were expressed as the expected change in the number of reported domestic human cases in SE.

## Materials and Methods

Data on alternative salmonella control strategies was available from two European countries, Denmark (DK) and the Netherlands (NL) (unpublished data). In the present paper, these countries were therefore used for comparison with the current SE control programme. The hypothetical implementation in SE of these two control strategies is hereafter called the DK and NL scenarios. It was assumed that if the current salmonella control programme in SE would be replaced by the salmonella control measures currently in place in DK or NL, the domestic exposure of humans to salmonella in SE would become equal to the level of domestic exposure in DK or NL, respectively. Due to differences in health care seeking behaviour and sensitivity of the surveillance system, the incidences of reported cases in different countries are not comparable [5], [6]. As truly comparable data are lacking, we used data from five different sources to try to obtain comparable estimates of exposure to salmonella in the different countries. Using these five data sources and given the hypothetical implementation of the two alternative control programmes in SE, the expected numbers of reported human cases in Sweden in 2010 in the different scenarios were estimated. Then the expected numbers of reported human domestic cases in Sweden in 2010 in the different scenarios were estimated using two different methods. In the first method it was assumed that the proportion of domestic cases in SE would be the same as in DK (in the DK scenarios) and as in NL (in the NL scenarios). In the second method it was assumed that the number of travel related cases reported in SE in 2010 (*n* = 2 764, Table 1) would remain constant. Finally, the differences between these estimates and the number of human domestic cases that was actually reported in Sweden in 2010 were estimated. The different data sources and calculations are introduced briefly below. Input data are detailed in table 1 and details of the calculations are provided in Appendix S1. In the text, the term scenario (singular) is defined as implementing a control strategy from DK or NL and specifying the data source used to estimate the change in the number of cases of salmonella and the method to estimate the number of domestic cases. Thus DK/1b is a scenario where the DK control strategies were implemented in SE, and where the number of reported cases of human salmonella in SE was estimated using data source 1 (sero-incidence data, see below) and where the number of reported domestic cases was calculated assuming that the number of travel related cases in SE would be constant (method b). Given that the change in the salmonella control programme would occur instantly, the expected change in salmonella exposure was also assumed to occur at once.

**Table 1:** Input values used to estimate the expected change in the number and incidence (cases/100 000 inhabitants) of reported domestic human salmonella cases in Sweden in 2010 in the different scenarios.

Input values	SE	DK	NL	References
Total number (incidence) of reported cases (2010)	3 609 (38)	1 598 (28.7)	2 291*(8.8)	SE [3], DK [10], NL [11]
Proportion domestic of the total number of reported cases (2010)	23.4%	54.8%	90%	SE [3], DK [10], NL [11]
Number of reported domestic (travel related) cases in Sweden(2010)	845 (2764)			SE [3]
Population (2010)	9 408 320	5 560 628	16 654 979	SE [12], DK [13],NL [14]
Multiplier (data source 1)	134	289	1 064	[5]
Underdetection index UDI (data source 2)	1	1.8	7.7	[7]
Under-reporting factor URF (data source 3)	0.5	4.4	26.3	[8]
Multiplier (data source 4)	10	17	20	[6]
Multiplier (data 5)	6.7**	10***	16.6****	

* Laboratory surveillance data from RIVM NL covers only 64% of the population. Cases adjusted to cover whole population: 1466/0.64 = 2291.

** Sundström, K. (2010) [9].

*** Email. com. 15 November 2011, S. Ethelberg, Statens Serum Institut, DK.

**** Email. com. 2 April 2012, W. van Pelt, Rijksinstituut voor Volksgezondheid en Milieu, NL.

### The different data sources used in the study

#### Data source 1 – Sero-incidence data

In a study by Falkenhorst *et al.* [5], serological cross-sectional studies were done to compare infection risks in different European countries independent of the effect of under-diagnosis and under-reporting. Using Bayesian backcalculation methods, the sero-incidence (cases/1000 person years) of salmonella, mainly *S*. Typhimurium and *S*. Enteritidis, was estimated. Country specific multipliers (ratios between sero-incidence and reported incidence) for the same years were also estimated [5] (Table 1).

In the present study, it was assumed that the multipliers did not change over time. Using these multipliers and data on the reported total number of cases in SE, DK and NL in 2010, the expected numbers of sero-positive cases in these countries were estimated. Expected sero-incidences for 2010 were then calculated. After that, the ratios between the expected sero-incidences for 2010 for DK/SE and then for NL/SE were calculated, reflecting the relations in exposure to salmonella between the countries. The ratios were then multiplied with the total number of reported cases in SE in 2010. Thereby an estimate of the expected total number of reported cases in SE was obtained, assuming that the salmonella situation would become the same in SE as in DK or NL, respectively. To obtain the expected number of domestic cases in SE, it was assumed that the proportion of domestic cases in SE would be the same as in DK and NL, respectively (method a) or that the number of reported travel related cases in SE would remain constant (method b). Given these assumptions, the expected number of reported domestic cases in Sweden could be estimated for the DK/1a, DK/1b, NL/1a and NL/1b scenarios. Finally, using these estimates, the expected change in the number of reported domestic cases in SE in 2010 was calculated for the different scenarios. Corresponding figures, expressed as incidences, were also calculated.

#### Data source 2 - Travel data I

The second data source originates from a study by deJong *et al.* (2006) [7]. In that study, estimates of the true incidence of salmonella (cases/100 000 inhabitants) in different European countries were calculated, based on salmonella risk (cases/100 000 travellers) in SE travellers returning from these countries between 1997–2003. These estimates were considered to be unbiased, making a comparison of salmonella exposure between countries possible [7]. An under-detection index (UDI) for country *c* was calculated as the ratio between the estimated incidence and reported incidence for country *c* divided by the ratio for Norway [7] (Table 1). As the study was based on SE data, no UDI could be calculated for Sweden and therefore the UDI for Sweden was assumed to be the same as for Norway [7]. As in data source 1, UDI was assumed to be constant over time. The calculations of the expected change in the number of reported domestic Swedish cases in the different scenarios were done in a similar way as for data source 1.

#### Data source 3 - Travel data II

True incidence data have also been calculated based on disease risks in returning Swedish travellers for the years 2005–2009 in Havelaar *et al.* [8]. An under-reporting factor (URF) was calculated in a similar way as in de Jong *et al.* [7], [8]. However, in Havelaar *et al.* [8], risks were expressed as relative to the risk of travelling to the Netherlands instead of to Norway as was the case in deJong *et al.* [7], [8]. Furthermore, as in the study by deJong *et al.* [7], the incidence rate could not be calculated for Sweden so the figure for Finland was used instead in Havelaar *et al.* [8]. In the present study, the UDIs from the study by Havelaar *et al.* [8] (Table 1) were used to calculate the expected change in the reported number of domestic salmonella cases in Sweden in a similar way as in previous calculations.

#### Data source 4 - Reconstruction of the surveillance pyramid

True incidence estimates to be used for international comparison have been estimated by reconstructions of the surveillance pyramids of seven European countries [6]. The study was based on surveillance data from 2001–2005 and additional survey data from 2008–2009. Using data on the health care systems, information on pathogen characteristics that influence health care seeking and information on health care usage obtained from harmonized cross sectional studies, the degrees of under-diagnosis and under-reporting for the seven European countries were estimated and expressed as multipliers (Table 1).

In the present study, these multipliers were used to calculate the expected increase in cases in SE in a similar was as in previous calculations.

#### Data source 5 - Expert opinion and reconstruction of the surveillance pyramid

Multipliers from the fifth data source were based on expert opinion. In DK, the multiplier was estimated to be between 5 and 20 with a most likely value of 10 to 20 (mail. com. S. Ethelberg, Statens, Serum Institut, DK, 15 November 2011). In the present study, a conservative estimate, a multiplier of 10 was used for DK. In NL, the multiplier was estimated to be 16.6 (mail. com. W. van Pelt, Rijksinstituut voor Volksgezondheid en Milieu, NL, 2 April 2012). In Sweden the multiplier was estimated to be 6.7, based on a reconstruction of the reporting pyramid [9]. In the present study, these multipliers were used to estimate the expected increase in cases in SE in a similar way as in previous calculations.

### Comparison of underreporting factors

In order to facilitate for the reader to compare the URF/UDI/multipliers used in the present study, an additional calculation was made where they were normalized, *i.e.* divided by the multipliers for SE. The normalized multipliers reflect the relation between the underreporting in SE and in DK and NL in the five data sources.

## Results

The estimated reported number (and incidence) of salmonella cases in SE in 2010 in the ten different scenarios are detailed in table 2. For example, using data source 1 and assuming that the SE programme would become similar to the DK programme, the total number of reported salmonella cases in SE in 2010 was estimated to be 5 831. The actual number of total reported cases in 2010 was 3 609 (Table 1).

**Table 2:** Estimation (number and incidence [cases/100 000 inhabitants]) of the total number of reported salmonella cases in Sweden in 2010, in the different scenarios.

Data sources	DK scenarios		NL scenarios	
	Number	Incidence	Number	Incidence
1. Sero-incidence	5 831	62	1 0276	109
2. Travel data I	4 867	52	9 965	106
3. Travel data II	23 793*	253*	68 074*	724*
4. Reconstruction of the surveillance pyramid	4 596	49	2 585**	28**
5. Expert opinion/reconstruction of the surveillance pyramid	4 503	48	3 452**	37**

* Values considered as unrealistically high.

** Values considered to be unrealistically low.

The final results, the expected increases in the number (and incidences) of reported domestic human cases in 2010 SE for the different scenarios, are detailed in tables 3 and 4. As an example, using data source 1 and method b and assuming that the SE programme would become similar to the DK programme, the expected increase in the number of reported domestic salmonella cases in SE in 2010 was estimated to be 2 222. Results of this calculation for scenarios DK/3, NL/3, NL/4 and NL/5 were not included as the estimated reported number of salmonella cases in SE in 2010 (Table 2) were considered to be unrealistic.

**Table 3:** Expected increase in the number of reported domestic salmonella cases in Sweden in 2010, if the Swedish salmonella control had been substituted with a programme similar to the Danish (DK) or the Dutch (NL) control programme.

Data source	Method a		Method b	
	DK scenarios	NL scenarios	DK scenarios	NL scenarios
1. Sero-incidence	2 351 (DK/1a)	8 404 (NL/1a)	2 222 (DK/1b)	6 667 (NL/1b)
2. Travel data I	1 822 (DK/2a)	8 124 (NL/2a)	1 258 (DK/2b)	6 356 (NL/2b)
3. Travel data II	*	*	*	*
4. Reconstruction of the surveillance pyramid	1 674 (DK/4a)	*	987 (DK/4b)	*
5. Expert opinion/reconstruction of the surveillance pyramid	1 623 (DK/5a)	*	894(DK/5b)	*

* Not included as the total number of cases (Table 3) was considered unrealistic.

Figures are given for each of the five data sources and for the two methods to estimate the number of domestic cases; percent domestic in DK and NL (method a) and number of reported travel related cases in SE in 2010 (method b).

**Table 4:** Expected increase in the incidence (cases/100,000) of reported domestic salmonella cases in Sweden in 2010, if the Swedish salmonella control had been substituted with a programme similar to the Danish (DK) or the Dutch (NL) control programme.

Data source	Method a		Method b	
	DK scenarios	NL scenarios	DK scenarios	NL scenarios
1. Sero-incidence	25 (DK/1a)	90 (NL/1a)	24 (DK/1b)	71 (NL/1b)
2. Travel data I	19 (DK/2a)	86 (NL/2a)	13 (DK/2b)	68 (NL/2b)
3. Travel data II	*	*	*	*
4. Reconstruction of the surveillance pyramid	18 (DK/4a)	*	10 (DK/4b)	*
5. Expert opinion/reconstruction of the surveillance pyramid	17 (DK/5a)	*	10 (DK/5b)	*

* Not included as the total number of cases (Table 2) was considered to be unrealistic.

Figures are given for each of the five data sources and for the two methods to estimate the number of domestic cases; percent domestic in DK and NL (method a) and number of reported travel related cases in SE in 2010 (method b).

The normalized multipliers obtained from the five data sources are detailed in table 5

**Table 5:** Normalized multipliers, *i.e.* the multipliers for Sweden, Denmark and The Netherlands from the five different data sources, divided by the multipliers for Sweden.

Data source	Normalized multipliers		
	Sweden	Denmark	Netherlands
1. Sero-incidence	1	2.16	7.94
2. Travel data I	1	1.80	7.70
3. Travel data II	1	8.80	52.60
4. Reconstruction of the surveillance pyramid	1	1.70	2.00
5. Expert opinion/reconstruction of the surveillance pyramid	1	1.67	2.67

## Discussion

Although other factors than the SE salmonella control programme (control in the whole food chain and import control of food of animal origin) can affect the domestic salmonella exposure, such as differences in eating habits, the control programme is considered to be the most important factor for the low domestic salmonella exposure in SE. The assumption that a relaxation of the Swedish control programme would affect the human exposure to salmonella seems reasonable.

There were three main reasons for using DK and NL to analyse the potential implications of a relaxation of the SE salmonella control programme. Firstly, human and animal populations in DK and the NL are reasonably comparable with SE with regard to societal aspects, production structures, climatic conditions etc. Secondly, sufficient data was available from these countries to enable all calculations. Thirdly, the salmonella situation in these countries is more similar to SE than most other European countries.

Comparing reported incidences of salmonella among countries, also within the European Union, can be very misleading [5], [6]. Therefore other data sources were needed to estimate differences in domestic exposure for salmonella in the countries. UDI, URF or multipliers (hereafter named multipliers) may be used to obtain estimates of salmonella exposure (data source 1) or salmonella infections (data sources 2–5) that are comparable between countries. In the present study, multipliers were used to estimate the relation between salmonella exposure or infections in the different countries. The multipliers obtained from the five data sources were calculated based on data from earlier years, but they were assumed to be constant over time and could thus be used in this present study together with data from 2010. Country incidence may change over time, as exemplified by the decrease in DK [10], however, the multipliers, reflecting country specific under-diagnosis and under-reporting, are expected to be more stable over time. A similar assumption seems to have been made by Havelaar *et al.* [8] where under-reporting factors for 27 EU-member states were calculated based on Swedish travel data for years 2005–2009 and used on data for 2009. In the present study, the multipliers were used with the number of reported salmonella cases from 2010 to estimate the relation between salmonella exposure in the different countries. An alternative would have been to use the number of reported salmonella cases for several years, as in Haagsma *et al.* [6]. However, including previous years for DK, when the domestic incidence was higher, would have overestimated the increase in salmonella cases in the DK scenarios. The calculations are therefore based on the situation in 2010, the last year from which complete data were available at the time the study was initiated.

Multipliers from the first data source were ratios between the estimated sero-incidence and reported incidence in SE, NL and DK [5]. The estimated sero-incidences have been shown to be correlated with infection risks in Swedish travellers described in the study by deJong *et al.* [7] but had a trend towards inverse correlation with reported cases [5]. These data were considered to be more appropriate for comparison between countries than reported incidence [5]. The back calculation used to obtain sero-incidence utilizes an antibody decay curve based on Danish data [5]. In high incidence countries with frequent infections this could result in an overestimation of the sero-incidence compared to the (unknown) true incidence of salmonella infections [5]. It cannot be excluded that sero-incidence may have been overestimated in NL and that this would result in an overestimation of cases in the NL/1 scenario in the present study. However, as NL is not a high-incidence country, this is not considered to substantially affect the output of this study.

Multipliers from the second data source were obtained from the study by deJong *et al.* [7], where it was concluded that the calculated UDI can be used for an unbiased comparison of salmonella exposure in humans. As the method was based on SE data, no UDI could be calculated for SE and therefore the UDI for Norway was used [7]. Using this UDI in the present study was considered reasonable as the salmonella situation and the surveillance system in Norway is similar to SE. The relation between normalized multipliers from data source 2 in the three countries were very similar to those from data source 1 (Table 5), which may be expected as Falkenhorst *et al.* [5] has shown that their results correlated well with results in deJong *et al.* [7].

Multipliers from data source 3 originate from a more recent study by Havelaar *et al.* [8] that also uses salmonella risks in returning SE travellers and a similar method as in de Jong *et al.* [7] to calculate true incidences and under-reporting of salmonella across the EU. In contrast to the study by deJong *et al*, [7] where cases were anchored to data reported by Norway where no underreporting was assumed to occur, in the study by Havelaar *et al.* [8] the estimates were anchored to a Dutch population based survey resulting in different URF [8]. The incidence rates for Sweden were assumed to be the same as in Finland resulting in a multiplier that was less than one which was considered to be highly unlikely, as it would mean that more cases are reported than actually exist [8]. When using this multiplier in the present study, the estimated total number of cases in the various scenarios became unrealistically high (Table 2). Even if the multiplier value for Sweden was raised to one, the increase in the number of reported domestic cases was still unrealistically high (results not shown). As it was not possible to estimate the correct incidence rates for Finland (and thereby Sweden) in the study by Havelaar *et al.* [8] and since the results based on this study was considered to be unrealistic, further calculations were not done using these data.

Multipliers from the fourth data source originated from a study aiming at developing a transparent model to reconstruct the surveillance pyramid for seven EU member states for seven pathogens that causes gastroenteritis [6]. Estimates were based on harmonized questionnaires but also on previous studies and expert opinions. The authors conclude that the degree of under-reporting and under-diagnosis varies greatly between countries and that the main factor that causes these discrepancies is differences in health care usage. However, it is also highlighted that there is considerable uncertainty, which may, at least in part, be due to uncertainties in the parameters used when reconstructing the surveillance pyramid [6]. Additionally, the surveys that were performed did not distinguish between viral and bacterial gastroenteritis, where the former has a milder and shorter course. This may have lead to an inflation of the multipliers [6]. The authors conclude that despite uncertainties the estimated incidences are a better basis for comparing disease incidences in different countries than official statistics. However, when using these data, the estimated incidence of reported salmonella cases in SE in the NL/4 scenario was 28/100 000 which is lower than the actually reported incidence in SE in 2010, 38/100 000 (Table 1). This was considered very unlikely as the study by Falkenhorst *et al.* [5] has shown that the exposure for salmonella in the NL population was higher (about three times) compared to the SE population. The reason that the estimates in the present study are so low is that the reported incidence in NL is much lower compared to Sweden, 8.8 and 38 cases per 100 000 inhabitants respectively (Table 1) and that the normalized multiplier is lower in data source 4 than in data sources 1, 2 and 3 (Table 5).

Multipliers from data source 5 were based on expert opinion. However, as with data source 4 the multiplier for NL was low (Table 5) resulting in the same unlikely results.

In the present study the number (and incidence) of cases that would be of domestic origin was estimated in two ways. Firstly it was assumed that the proportion of domestic cases in SE would be the same as the proportion of domestic cases in DK and NL, respectively. The proportion of domestic cases depends on domestic exposure of salmonella in the country and it seems reasonable that the proportion of domestic cases is higher in a country where the domestic exposure to salmonella is higher. Therefore is seems reasonable that the proportion of domestic cases would increase if the domestic exposure to salmonella in SE became similar to DK or NL. However, other factors could also affect the proportion of cases being domestic, such as differences in the number of travellers and their incidence of salmonella. Furthermore differences in health care seeking behaviour and differences in the likelihood of having samples taken after travelling as well as eating habits can affect the proportion of cases being domestic. However, as the proportion of cases reported to be domestic was very high in NL (90%), the number (and incidence) of cases that would be domestic was also calculated assuming that the actual number of travel related cases that was reported in SE in 2010 (*n* = 2 764) would not change given a relaxation in the Swedish salmonella control programme.

In the present study it was also assumed that the domestic exposure would change instantly after a relaxation of the control. The requirement for testing of imported animal products (the additional guarantees) would cease, leading to an increased salmonella prevalence in imported food of animal origin. At present in SE, salmonella in imported food is considered to be an important source for salmonella in humans [15] and this would be expected to increase even more. Furthermore, as the prevention and eradication strategy of salmonella in pig production would cease, any introduction of salmonella would spread rapidly in the pig population due to the production system. In the NL scenario, the same applies for the poultry production. Salmonella in cattle production would probably spread more slowly, but cattle is not considered to be among the major sources of salmonella for humans [16]. The indirect exposure of humans from environmental contamination originating from food animal production would be expected to increase successively and not at once. In conclusion, as the major part of the expected increased exposure would occur soon after any change of the programme, this assumption was considered reasonable.

As comparable data of salmonella exposure in humans in different countries do not exist, the present study focuses on the possibility of using multipliers to obtain estimates of salmonella exposure that are comparable between countries. These estimates were then used to calculate the expected increase in the number of reported domestic cases in SE. It can be concluded that, apart from scenarios based on the study by Havelaar *et al.* [8], the expected increase of domestic salmonella cases in the DK scenarios were of the same magnitude, *i.e.* between 894 and 2 351 cases (Table 3). For the NL scenarios, the expected increase in cases varied between 6 356 and 8 404 (Table 3).

Variability and uncertainty was not quantified in the present study due to a lack of data. However for data source 1 and 2 an attempt was still made to estimate the variability and uncertainty in expected reported number of domestic cases in SE, using Pert distributions and running 5 000 iterations in @Risk (Palisade Co.). In these simulations it was assumed that the number of travel related cases would be constant (results not shown). In the two DK data sources, the lowest 5% percentile was below 0 indicating a decrease in these scenarios. This is highly unlikely, as Falkenhorst *et al.* (2012) have shown that the exposure for salmonella is higher (about 50%) in DK than in SE. The highest 5% percentile was 5603. Corresponding figures for NL were 3621 and 13126. However, these figures give an impression of precision in the estimates that does not exist. It was concluded that there is a high degree of uncertainty in the data sources. We therefore consider it more robust to use the expected value of the different scenarios to support our conclusions and highlight that considerable uncertainty exists and that it was not possible to quantify uncertainty and variability in a reliable way.

The present study supports statements by others [5], [6], [7], [8] that comparable data on the exposure to salmonella and other pathogens are needed. Concerning the animal populations, well designed and harmonized baseline studies have been done in EU for broilers, layers, turkeys, fattening pigs as well as for breeding pigs [17], [18], [19], [20], [21]. Such studies are needed to evaluate the effects of control measures implemented on EU level. However, as the reason for controlling salmonella in animals is to decrease salmonella exposure to humans, the effect of implemented control measures should preferably be evaluated by baseline studies in humans. Pending such studies, results of serological testing [5] is promising and should, once validated, be repeated on sera from more recent years. More studies are also needed to clarify the relation between sero-incidence and the true number of salmonella cases [5].

The main conclusion from the present study is that the number of reported domestic cases in Sweden is expected to increase with some 1–2 thousand cases in the DK scenarios and more in the NL scenarios. This increase would partly be caused by an increase in the salmonella prevalence in domestically produced food of animal origin. However, a more important source would probably be salmonella contaminated imported food, as SE would no longer be allowed to require salmonella testing prior to import. In the future, if the salmonella prevalence continues to decrease due to extended common EU control programmes for salmonella [1], the effect for Sweden of losing its additional guarantees (including salmonella control of imported food of animal origin) will decrease, and thereby the expected increase in the number of human cases, as estimated in this study, would be less. Similarly, if the domestic exposure to salmonella continues to decrease in DK and NL, the expected increase for SE as calculated in this paper would be lower. The present study should therefore be seen as an attempt to estimate what would have happened in SE if a relaxation in salmonella control had been implemented, using data from 2010.

## Supporting Information

Appendix S1 **Calculations to estimate the change in the number of reported domestic human salmonella cases in Sweden in the DK and NL scenarios, using five different data sources.**
(DOCX)

## References

[1] European Food Safety Authority, European Centre for Disease Prevention and Control (2012) The European Union. Summary Report on Trends and Sources of Zoonoses, Zoonotic Agents and Food-borne Outbreaks in 2010;. EFSA Journal 2012 10 (3) 2597 [442pp.] doi:10.2903/j.efsa.2012.2597. Available online: www.efsa.europa.eu/efsajournal

[2] Anonymous (2011) Surveillance of zoonotic and other animal disease agents in Sweden 2010 Available: http://www.sva.se/upload/Redesign2011/Pdf/Om%20SVA/publikationer/1/Zoonosrapport_2010_webb.pdf.Assessed 10 June 2012.

[3] Smittskyddsinstitutet (2011) Smittskyddsinstitutets epidemiologisk årsrapport 2010; 2010 [The annual epidemiological report from The Swedish Institute for Communicable Disease Control, 2010] Available: http://www.smi.se/upload/Publikationer/Epidemiologisk-%C3%A5rsrapport-2010-2011-1-5.pdf.Assessed 3 June 2012.

[4] Engvall A, Andersson Y, Cerenius F (1994) The economics of the Swedish Salmonella control- A cost-benefit analysis. NVI/WHO International course on Salmonella control in animal production and products, A presentation of the Swedish Salmonella programme. Malmö, Sweden.

[5] Falkenhorst G, Simonsen J, Ceper TH, van Pelt W, de Valk H, et al (2012) Serological cross-sectional studies on salmonella incidence in eight European countries: no correlation with incidence of reported cases. Bmc Public Health 12: 523. 22799896 10.1186/1471-2458-12-523 PMC3490876

[6] Haagsma JA, Geenen PL, Ethelberg S, Fetsch A, Hansdotter F, et al (2013) Community incidence of pathogen-specific gastroenteritis: reconstructing the surveillance pyramid for seven pathogens in seven European Union member states. Epidemiology and Infection 141 (8) 1625–39. 23013659 10.1017/S0950268812002166 PMC9151593

[7] de Jong B, Ekdahl K (2006) The comparative burden of salmonellosis in the European Union member states, associated and candidate countries. Bmc Public Health 6: 4. 16403230 10.1186/1471-2458-6-4 PMC1352352

[8] Havelaar A, Ivarsson S, Löfdahl M, Nauta M (2012) Estimating the true incidence of campylobacteriosis and salmonellosis in the European Union, 2009. Epidemiology and Infection 1: 1–10. 10.1017/S0950268812000568 PMC9152072 22717051

[9] Sundström K (2010) Folkhälsa - Djurhälsa, Ny ansvarsfördelning mellan stat och näring,SOU 2010:106, Del C, Bilaga 9 Samhällskostnader för salmonellos, campylobacterios och EHEC [Societal costs for salmonellosis, campylobacteriosis and EHEC]. Available: http://www.regeringen.se/content/1/c6/15/92/06/8777a1ea.pdf. Assessed 12 June 2012.

[10] Anonymous (2010) Annual Report on Zoonoses in Denmark 2010; Available: http://www.food.dtu.dk/upload/f%C3%B8devareinstituttet/food.dtu.dk/publikationer/tilbagevendende_publikationer/annual%20report%20on%20zoonoses/annual+report+2010.pdf.Assessed 22 May 2012.

[11] RIVM (2010) Rijksinstituut voor Volksgezondheid en Milieu (RIVM), Staat van Zoönosen 2010; Available: http://www.rivm.nl/bibliotheek/rapporten/330291007.pdf.Assessed 10 June 2012.

[12] Anonymous. Statistics Sweden. Available: www.scb.se. Assessed 15 may 2012.

[13] Anonymous. Statistics Denmark. Available: www.dst.dk. Assessed 15 may 2012.

[14] Anonymous. Statistics Netherlands. Available: www.cbs.nl. Assessed 15 may 2012.

[15] Wahlstrom H, Andersson Y, Plym-Forshell L, Pires SM (2011) Source attribution of human Salmonella cases in Sweden. Epidemiology and Infection 139: 1246–1253. 20943003 10.1017/S0950268810002293

[16] Pires SM, de Knegt L, Hald T (2011) National Food Institute, Technical University of Denmark. Estimation of the relative contribution of different food and animal sources to human Salmonella infections in the European Union, Scientific report/Technical report submitted to EFSA. Published July 2011. Available: http://www.efsa.europa.eu/en/supporting/pub/184e.htm.

[17] EFSA (2009) Analysis of the baseline survey on the prevalence of Salmonella in holdings with breeding pigs, in the EU, 2008, Part A: Salmonella prevalence estimates,. EFSA Journal 2009 7 (12) 93

[18] EFSA (2008) Report of the Task Force on Zoonoses Data Collection on the Analysis of the baseline survey on the prevalence of Salmonella in turkey flocks, Part A,. The EFSA Journal (2008) 134: 1–91.

[19] EFSA (2007) Report of the Task Force on Zoonoses Data Collection on the Analysis of the baseline survey on the prevalence of Salmonella in broiler flocks of Gallus gallus, Part A,. The EFSA Journal (2007) 98: 1–85.

[20] EFSA (2007) Report of the Task Force on Zoonoses Data Collection on the Analysis of the baseline study on the prevalence of Salmonella in holdings of laying hen flocks of Gallus gallus,. The EFSA Journal (2007) 97.

[21] EFSA (2008) Report of the Task Force on Zoonoses Data Collection on the analysis of the baseline survey on the prevalence of Salmonella in slaughter pigs, Part A,. The EFSA Journal (2008) 135: 1–111.

