# Supplementary material for: Estimation of the Expected Change in Domestic Human Salmonella Cases in Sweden in 2010, Given a Hypothetical Relaxation of the Current Salmonella Control Programme
Source: PLoS One. 2014 Mar 3;9(3):e89833. doi: 10.1371/journal.pone.0089833 (PMC3940613; doi:10.1371/journal.pone.0089833)
Supplement: Appendix S1 — Calculations to estimate the change in the number of reported domestic human salmonella cases in Sweden in the DK and NL scenarios, using five different data sources. (DOCX) [file pone.0089833.s001.docx]

# Appendix S

*Calculations to estimate the change in the number of reported domestic human salmonella cases in Sweden in the DK and NL scenarios, using five different data sources*

The calculation is based on sero-incidence data, however the principles are the same for all five data sources.
 The number of persons with antibodies to salmonella in the three countries (*c*) SE, DK and NL in 2010 (*SeroCases_c_*): was estimated by multiplying the number of reported cases in 2010 with a multiplier (*Multiplier_c_*).

*$SeroCases_{c}$=$RCases_{c}$ $\times$ $Multiplier_{c}$* (1)

where *RCases_c_* is the number of reported cases in country *c* in 2010. The expected sero-incidence for country *c* in 2010 (*ESeroI_c_*) was calculated as:

$ESeroI_{c}$=$SeroCases_{c}$ $\div$ $Pop_{c}$ (2)

where *Pop_c_* is the population in country *c* in 2010. The expected number of reported cases in SE in 2010 if the exposure to salmonella would be the same as in DK (*ERCases_SE(DK)_*) was calculated as

$ERCases_{SE(DK)}$=$RCases_{SE}$ $\times$($ESeroI_{DK}$ $\div$ $ESeroI_{SE}$) (3)

Where *RCases_SE_* is the number of reported cases in SE in 2010. *ESeroI_DK /_ ESeroI_SE_* is the ratio between expected sero-incidence for DK and SE, reflecting the increased risk of exposure to salmonella in SE in the DK scenario. The expected number of reported domestic cases in Sweden if the exposure to salmonella would be the same as in DK (*ERDCases_SE(DK)_*) was calculated in two different ways. First (method a) it was calculated as

$ERDCases_{SE(DK)}$=$ERCases_{SE(DK)}$ $\times$ $\text{PrRD}_{DK}$ (4)

where *PrRD_DK_* is the proportion of reported salmonella cases in DK in 2010 that was considered to be of domestic origin. Secondly (method b), the expected number of reported domestic cases in SE was calculated by subtracting the number of reported travel related salmonella cases in SE in 2010 (2 764) from the expected number of reported cases in SE in 2010 if the exposure to salmonella would be the same as in DK (*ERCases_SE(DK)_*). Finally the expected increase in number of reported domestic cases in SE in 2010 if the exposure to salmonella became the same as in DK (*IncrERDCases_SE(DK)_*) was calculated as

$IncrERDCases_{SE(DK)} =ERDCases_{SE(DK)} -RDCases_{SE}$ (5)

where *RDCases_SE_* is the number of reported domestic cases of salmonella in SE in 2010. The expected increase in number of reported domestic cases in SE if the exposure to salmonella became the same as in NL was calculated in the same way.
